# Rapid senescence‐like response after acute injury

**DOI:** 10.1111/acel.13201

**Published:** 2020-08-02

**Authors:** Xiaogang Chu, Jin Wen, Raghavan Pillai Raju

**Affiliations:** ^1^ Department of Pharmacology and Toxicology Medical College of Georgia Augusta University Augusta GA USA

**Keywords:** acute senescence, aging, cell cycle, senescence‐associated secretory phenotype

## Abstract

Cellular senescence is a state of irreversible growth arrest. Short‐term programmed senescence such as in embryonic development and slowly progressing senescence as in aging are both well described. However, acute senescence in living organisms is not well understood. We hypothesized that hemorrhagic shock injury (HI) caused by whole body hypoxia and nutrient deprivation, resulting in organ dysfunction due to severe blood loss, could lead to acute senescence in vivo. Our experiments, for the first time, demonstrate a rapidly emerged, senolytics‐responsive, senescence‐like response in the rat liver in less than five hr following hemorrhagic shock. We conclude that the senescence, or pseudosenescence, observed is necessary to maintain tissue homeostasis following the injury.

## Graphical abstract

In this study, we show that rats subjected to hemorrhagic shock, an acute injury, develop a senescence‐like response within a few hours. Treatment of these animals with senolytics reduced survival, suggesting that the senescence, or pseudosenescence, observed may be necessary for tissue homeostasis following the injury.

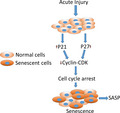

## 1 INTRODUCTION, RESULTS AND DISCUSSION

Cellular senescence is a process in which cells enter a state of stable cell cycle arrest (Campisi, 2013). In 1961, Hayflick and Moorehead described the “degeneration” of in vitro cultured cells after finite divisions as “a phenomenon attributable to intrinsic factors which are expressed as senescence” (Hayflick & Moorhead, 1961; van Deursen, 2014). It is now known that senescence may be triggered or accelerated by excessive stress, including DNA damage, activation of oncogenes, mitochondrial dysfunction, or oxidative stress (Muñoz‐Espín & Serrano, 2014). Hemorrhagic shock injury (HI) due to the loss of a large volume of blood accounts for 30%–40% of trauma‐related deaths. A whole body insult due to hypoxia and nutrient deficiency is a characteristic of hemorrhagic shock and leads to a systemic inflammatory response and injury to multiple organs (Cannon, 2018; Poulose & Raju, 2014). Multiple stressors such as circulating DAMPS, oxidative stress, and mitochondrial dysfunction have been reported to be the important pathological hallmarks of hemorrhagic shock and the same factors are also reported to trigger or characterize senescence (Davalos et al., 2013; Herranz & Gil, 2018; Poulose & Raju, 2014).

In vivo models that describe features of senescence within a few hours following tissue insult or premature senescence following hemorrhagic shock have not been reported before. Based upon this premise we hypothesized that the acute injury caused by hemorrhagic shock will lead to a rapid development of senescence in vivo. Very limited information is available on rapid onset senescence in in vivo models. In this study, we tested whether hemorrhagic shock is characterized by acute senescence as there are several common metabolic denominators between hemorrhagic shock and aging phenotype (Gomes et al., 2013; Poulose & Raju, 2015).

**FIGURE 1:**
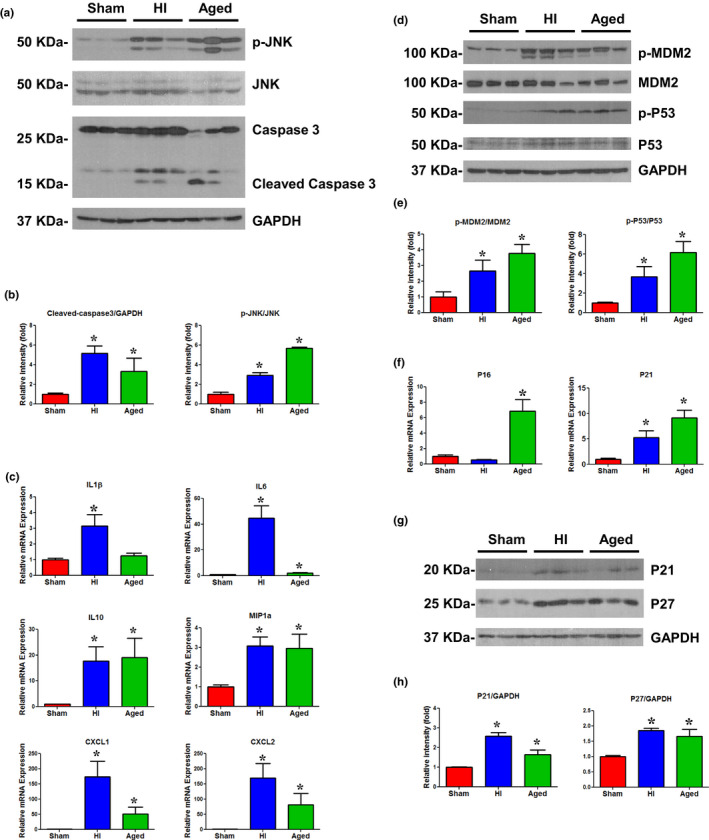
(a) Representative immunoblotting of p‐JNK, JNK, and Caspase3 in liver tissues of sham, HI, and aged rats. Loading control: GAPDH. (b) p‐JNK, JNK, cleaved‐Caspase3, and GAPDH band intensities (see Figure 1a) were quantified using Image J software (NIH). *n* = 6 animals/group, 2–3 technical replicates. *p < 0.05 vs. sham. (c) SASP gene expression was measured by real‐time PCR in the liver tissues of sham, HI, and aged rats. *n* = 5–6/group; two technical replicates; bars: mean ± *SEM*; *p < 0.05 vs. sham. (d) Representative immunoblotting of p‐MDM2, MDM2, p‐P53, and P53 in liver tissues of sham, HI, and aged rats. Loading control: GAPDH. (e) p‐MDM2, MDM2, p‐P53, P53, and GAPDH intensities (see Figure 1d) were quantified using Image J software (NIH). *n* = 6 animals/group, 2–3 technical replicates. *p < 0.05 vs. sham. (f) p16 and p21 gene expressions measured by real‐time PCR in the liver tissues of sham, HI, and aged rats. *n* = 5–6/group; two technical replicates; bars: mean ± *SEM*; *p < 0.05 vs. sham. (g) Representative immunoblotting of p21 and p27 in liver tissues of sham, HI, and aged rats. Loading control: GAPDH. (h) P21, p27, and GAPDH intensities (see Figure 1g) were quantified using Image J software (NIH). *n* = 6 animals/group, 2–3 technical replicates. *p < 0.05 vs. sham

We found a significant elevation in cleaved caspase‐3 and p‐JNK (*p* < 0.05) in the liver of mature rats subjected to HI as compared to mature sham operated rats (Figure 1a,b). These injury markers were similarly increased in the liver of aged rats. We further tested a panel of senescence‐associated secretory phenotype (SASP) markers by real‐time PCR in the livers of mature rats, aged rats, and mature rats subjected to HI. The cytokines IL‐1β, IL‐6, and IL‐10 and chemokines MIP‐1α, CXCL1, and CXCL2 demonstrated a significant (*p* < 0.05) increase in expression following HI (Figure 1c). The liver tissues from aged rats also showed a similar increase in SASP as in the rats subjected to HI, though the increase in IL‐1β was not significant.

**FIGURE 2:**
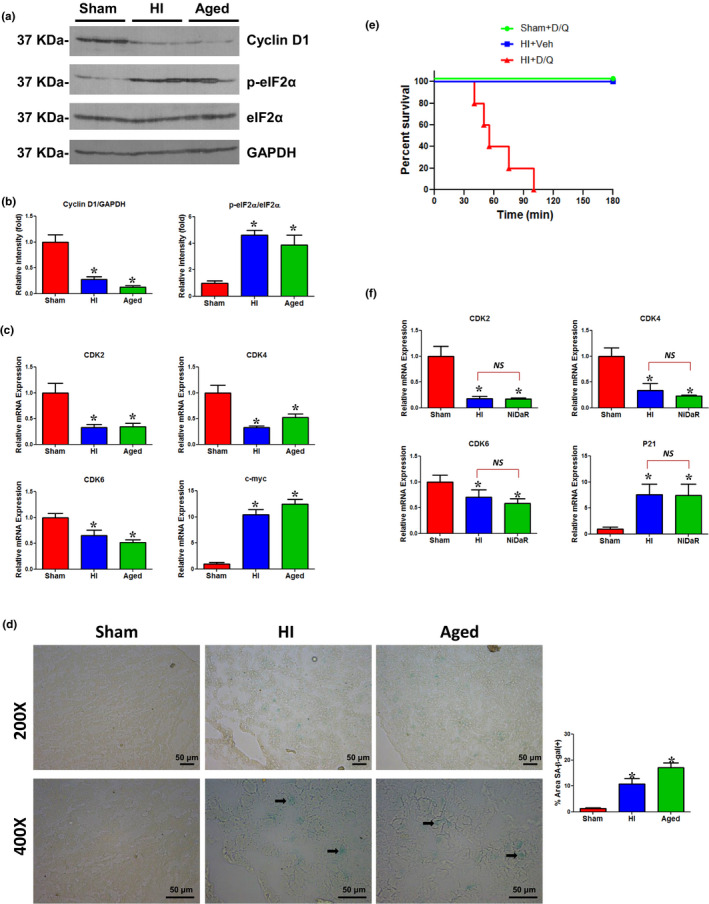
(a) Representative immunoblotting of Cyclin D1, p‐eIF2α, and eIF2α in liver tissues of sham, HI, and aged rats. Loading control: GAPDH. (b) Cyclin D1, p‐eIF2α, eIF2α, and GAPDH intensities (see Figure 2a) were quantified using Image J software (NIH). *n* = 6 animals/group, 2–3 technical replicates. *p < 0.05 vs. sham. (c) CDK2, CDK4, CDK6, and c‐myc gene expressions were measured by real‐time PCR in the liver tissues of sham, HI, and aged rats. *n* = 5–6/group; two technical replicates; bars: mean ± *SEM*; *p < 0.05 vs. sham. (d) Representative images of SA‐β‐gal staining the frozen liver sections from sham, HI, and aged rats. Manufacturer’s protocol (Cell Signaling, Danvers, MA) was used for SA‐β‐gal staining. Briefly, frozen sections were fixed for 15 min at room temperature, washed three times with PBS, and incubated with SA‐β‐gal staining solution overnight at 37 °C. Scale bars: 50 μm. Bar diagram on the right: SA‐β‐gal quantified by ImageJ software (NIH; Cai et al., 2020). Three animals/group. *p < 0.05 vs. sham. (e) Kaplan–Meier curves show survival rate in HI + Veh (*n* = 5), sham + D + Q (*n* = 5), HI + D + Q (*n* = 5) treated rats. (f) CDK2, CDK4, CDK6, and p21 gene expressions were measured by real‐time PCR in the liver tissues of sham, HI, and NiDaR (2 mg/kg of each of Niacin, Dichloroacetate, and Resveratrol) treated rats. *n* = 5–6/group; two technical replicates; bars represent mean ± *SEM*; NS, not significant, *p < 0.05 vs. sham group

As a major mediator of the DNA damage pathway, the p53/MDM2 axis has been shown to be critical for stress‐induced cellular senescence (Wu & Prives, 2018). We observed a significant elevation in the level of phosphorylated p53 and phosphorylated MDM2 in the liver of rats subjected to HI, as well as in the liver from aged rats (Figure 1d,e). To further test the induction of senescence in the liver following HI, gene and protein expression of several senescence markers were examined. Our experiments show that p21 and p27, but not p16, were increased in the liver following HI, suggesting that p21 and p27 may trigger senescence following HI (Figure 1f–h). However, in aged rats, there was a significant upregulation of not only p21 and p27 but also p16. The lack of upregulation of p16 following HI is consistent with the findings that p16 is a late stage marker (van Deursen, 2014). The p21 and p27 are cell cycle inhibitors and can arrest the cell cycle progression in G1/S and G2/M transitions by inhibiting CDK4, 6/cyclin D, and CDK2/cyclin E (Abukhdeir & Park, 2008). As shown in Figure 2a,b, there was a decreased expression of cyclin D1 and an elevated level of p‐eIF2α demonstrating cell cycle arrest in the liver following HI. The expression of cell cycle regulators CDK2, CDK4, and CDK6 was decreased when c‐Myc was markedly increased in both HI and aged rats (Figure 2c). The pharmacological inhibition of CDK2 has been shown to induce Myc‐dependent senescence (Campaner et al., 2010). The expression of the same markers in livers from aged rats and HI showed similar trends. Cellular senescence is usually accompanied by morphological changes and characterized by markers including increased activity of senescence‐associated β‐galactosidase (SA‐β‐gal) activity (Dimri et al., 1995). The liver sections from HI‐induced rats and normal aged rats displayed cellular staining for SA‐β‐gal further establishing a rapid onset of senescence following HI (Figure 2d).

The role of senescence in the pathological process of HI remains unknown. In order to test whether the rapid and newly emerged senescence‐like process following the acute injury is detrimental or beneficial to the outcome following HI, separate groups of rats were administered the combination senolytics, Dasatinib and Quercetin (D + Q), or vehicle. They were given as a single dose during fluid resuscitation, immediately after hemorrhage and shock. D + Q has been described as a senolytic and has been successfully tested in mice and humans to remove senescent cells (Hickson et al., 2019; Wissler Gerdes, Zhu, Tchkonia, & Kirkland, 2020). Surprisingly, all five animals that received D + Q, but not vehicle, died within the two hour observation period (range 40–100 min; Figure 2e). The senolytics‐mediated death of senescent cells is well described (Pignolo, Passos, Khosla, Tchkonia, & Kirkland, 2020; Zhu et al., 2015). The results suggest that the induction of acute senescence may be a necessary process in hemorrhagic shock. It is noteworthy that none of the sham operated animals that received D + Q died. The hemorrhagic shock‐induced tissue injury may be further exacerbated due to cell death mediated by senolytics resulting in the fatality of the animals. It is also possible that the compounds may have off‐target effects. As D is a tyrosine kinase inhibitor, its potency with respect to other molecular pathways cannot be ignored in acute injury conditions, as opposed to its safe use in age‐associated senescence. To further confirm whether the senescence markers are altered when pharmacological interventions improve organ function after HI, we tested rats treated with NiDaR (niacin, dichloroacetate, and resveratrol), a drug combination that improved organ function and survival following HI (Chu, Schwartz, Diamond, & Raju, 2019). The protocol used for the HI procedure and NiDaR treatment was identical to the one used for the D + Q treatment (Chu et al., 2019). The NiDaR treatment after HI did not inhibit the emergence of a senescence‐like molecular process in the liver, as we found the levels of CDK2, CDK4, CDK6, and p21 to be similar to the levels in the livers of animals subjected to HI without NiDaR treatment (Figure 2f). These experiments demonstrate that the rapid emergence of senescence is likely not harmful but may be protective in the outcome following HI. However, further studies are needed to determine the long‐term survival and functional significance of the newly emerged senescent cells.

In summary, our study shows for the first time that cellular senescence can develop in vivo within a few hours of insult to the tissue. We also demonstrated that HI led to the development of a senescence phenotype. Our experiments with the senolytics and NiDaR show that the rapidly developed senescence is not detrimental to the outcome following HI, rather it may have a protective role. The senescence process observed is likely a transient homeostatic response to injury and may be called pseudosenescence.

## CONFLICT OF INTEREST

None of the authors have any competing interest.

## AUTHOR CONTRIBUTIONS

XC and RR analyzed, interpreted, and drafted the manuscript. JW and XC performed the experiments. XC, JW, and RR finalized the manuscript.

## Supporting information

Supplementary Material

## Data Availability

Data that support the findings of this study are available from the corresponding author upon reasonable request.
