# Supplementary material for: Rapid senescence‐like response after acute injury
Source: Aging Cell. 2020 Aug 2;19(9):e13201. doi: 10.1111/acel.13201 (PMC7511876; doi:10.1111/acel.13201)
Supplement: Supplementary file 1 — Supplementary Material [file ACEL-19-e13201-s001.docx]

**Supplementary data (X. Chu et al)**

**Animal Study**

The animal experiments described in the study were approved by the Institutional Animal Care and Use Committee (IACUC) at Augusta University and were performed in accordance with the relevant guidelines and regulations. Male Sprague Dawley rats (10-12 weeks old) were obtained from Charles River Laboratory (Wilmington, MA, USA). These animals were used as sham controls or for HI procedure with/without senolytic treatment. Aged (22 month-old) Sprague Dawley rats were obtained from NIA rodent colony.

The animals were subjected to sham or HI procedure as described before (15). Briefly, the animals were anesthetized with isoflurane, a midline laparotomy (2 cm) was performed to induce soft tissue trauma and femoral arteries and vein cannulated. Soft tissue injury was induced to mimic tissue injuries in severe hemorrhage. One femoral artery was connected to a blood pressure analyzer, bleeding was performed through the other artery. The animals in the HI groups were bled 60% of the total blood volume rapidly to a mean arterial pressure (MAP) of 40 ± 5 mmHg. After 45 min of hemorrhage, the animals were maintained in the state of shock by maintaining the low MAP for another 45 min. Fluid resuscitation was carried out with Ringer lactate, twice the volume of shed blood. A combination of desatinib and quercetin (3 and 10 mg/kg respectively) were administered i.v. in 100 μl DMSO at 10 min after the start of resuscitation. Sham animals did not undergo hemorrhage or fluid resuscitation. The animals were euthanized at 2 h after the end of resuscitation and tissues saved. Historical specimens were used to test senescence in NiDaR treated rats (14).

**Statistics**

Data are presented as means ± S.E.M. of at least three separate experiments (each in triplicate or duplicate). Student t test or One-way ANOVA, was used for statistical analysis using Prism 6 (GraphPad Software). p<0.05 was considered to be statistically significant.

**Table S1. Primer sequences used.**

| **Gene** | **Forward primer sequence (5’-3’)** | **Reverse primer sequence (5’-3’)** |
| --- | --- | --- |
| IL6 | GAGCCCACCAGGAACGAAA | AACTGGCTGGAAGTCTCTTGC |
| IL1β | CCCTGCAGCTGGAGAGTGTGG | TGTGCTCTGCTTGAGAGGTGCT |
| IL10 | TGCGACGCTGTCATCGATTT | GTAGATGCCGGGTGGTTCAA |
| MIP-1α | CTGCCAAGTAGCCACATCCA | GGAATGTGCCCTGAGGTCTT |
| CXCL1 | GCCACACTCAAGAATGGTCG | TGGGGACACCCTTTAGCATC |
| CXCL2 | TCCTCAATGCTGTACTGGTCC | ATGTTCTTCCTTTCCAGGTC |
| CDK2 | AATCCGGCTCGACACTGAGACTG | CACGGTGAGAATGGCAGAATGCTAGGCCC |
| CDK4 | GCTACCACTCGATATGAACCCGTGGCTGAA | GGTGCTTTGTCCAGGTATGTCCGTAGGTCC |
| CDK6 | TCGTGGAAGTTCAGACGTGG | TTGCCTAGCTCGTCGATGTC |
| c-Myc | AAAGGCCCCCAAGGTAGTTA | CTCGCCGTTTCCTCAGTAAG |
| P16ink4a | TCGTGCGGTATTTGCGGTAT | TAGTCTCGCGTTGCCAGAAG |
| P21CIP1 | CAGCCACAGGCACCATGTC | ACAGACGACGGCATACTTTGC |
| β-actin | AGTACCCCATTGAACACG | AATGCCAGTGGTACGACC |

**Table S2. Antibodies used in this study.**

| **Antibodies for Western Blot** | **Host species** | **Dilution** | **Source** | **Catalogue** |
| --- | --- | --- | --- | --- |
| Phospho-SAPK/JNK (Thr183/Tyr185) | Rabbit | 1:1000 | Cell Signaling | 4668 |
| JNK1 | Mouse | 1:1000 | Cell Signaling | 3708 |
| Phospho-eIF2α (Ser51) | Rabbit | 1:1000 | Cell Signaling | 3398 |
| eIF2α | Rabbit | 1:1000 | Cell Signaling | 5324 |
| p27 Kip1 | Rabbit | 1:1000 | Cell Signaling | 3686 |
| GAPDH | Rabbit | 1:5000 | Cell Signaling | 2118 |
| P53 | Mouse | 1:1000 | Cell Signaling | 2524 |
| Caspase3 | Rabbit | 1:1000 | Cell Signaling | 9662 |
| Cyclin D1 | Rabbit | 1:1000 | Cell Signaling | 2978 |
| Phospho-MDM2 (Ser166) | Rabbit | 1:1000 | Cell Signaling | 3521 |
| p21 Waf1/Cip1 | Rabbit | 1:1000 | Cell Signaling | 2947 |
| Anti-p53 (phospho S15) | Rabbit | 1:1000 | Abcam | ab1431 |
| MDM2 | Mouse | 1:1000 | Santa Cruz Biotechnology | Sc-965 |
